# SLICC-Frailty Index and Its Association with Low Bone Mineral Density and Vertebral Fractures in Women with Systemic Lupus Erythematosus

**DOI:** 10.1007/s00223-023-01117-9

**Published:** 2023-07-23

**Authors:** Claudia Mendoza-Pinto, Ivet Etchegaray-Morales, Pamela Munguía-Realpozo, Socorro Méndez-Martínez, Jorge Ayón-Aguilar, Francisco Arellano-Avendaño, Álvaro Joaquín Montel-Jarquín, Mario García-Carrasco

**Affiliations:** 1 Systemic Autoimmune Diseases Research Unit, Specialties Hospital UMAE- CIBIOR, Mexican Institute for Social Security, Puebla, Puebla Mexico; 2 Department of Rheumatology, Medicine School, Meritorious Autonomous University of Puebla, Puebla, Mexico; 3 Research in Health Coordination, Mexican Social Security Institute, Puebla, Mexico; 4 Health Research Medical Coordinator, Mexican Social Security Institute, Puebla Delegation, Puebla, Mexico; 5 Specialties Hospital, Centro Médico Nacional “Manuel Ávila Camacho”, IMSS, Puebla, Mexico

**Keywords:** Systemic lupus erythematosus, Frailty, Vertebral fractures

## Abstract

The Systemic Lupus International Clinics (SLICC)-Frailty Index (FI) is associated with adverse outcomes in systemic lupus erythematosus (SLE). However, to our knowledge, its association with bone mineral density (BMD) and vertebral fractures (VF), has not been investigated using a standardized methods. Our aim was to evaluate the relationship between frailty assessed by SLICC-FI, and BMD and VF in Mestizo women with SLE. Adult women were included in this cross-sectional study. Information concerning the risk factors for VF and BMD in the lumbar spine and total hip was acquired. SLICC-FI was assessed at baseline. A semi-quantitative method was utilized to evaluate the prevalence of VF on lateral thoracolumbar radiographs. Univariate and multivariate regression analyses were performed adjusting for age, body mass index (BMI), SLE duration, cumulative glucocorticoid dose, bisphosphonate use, and BMD measurements. We included 202 women with SLE (mean age [SD] = 43.3 [13.6] years). The mean (SD) SLICC-FI value was 0.14 (0.09). Eleven (5.4%) patients were categorized as robust, 62 (30.7%) as relatively less fit, 84 (41.6%) as least fit, and 45 (22.3%) as frail. Both univariate and multivariate models showed associations between frailty (defined as SLICC-FI > 0.21) and prevalent VF in the entire population (OR 5.76, 95% CI 2.53–13.12; *P* < 0.001) and in the premenopausal group (OR 4.29, 95% CI;* P* = 0.047). We also found an association between the SLICC-FI and low BMD. In conclusion, frailty assessed by SLICC-FI might be associated with VF and low BMD in mestizo females with SLE.

**Supplementary Information:**

The online version contains supplementary material available at 10.1007/s00223-023-01117-9.

## Introduction

Vertebral fractures (VF) are more frequent in patients with systemic lupus erythematosus (SLE) than in the general population [1]. Patients with fractures may have decreased functional ability and a lower health-related quality of life (HRQL) [2–4]. Additionally, VF are associated with higher mortality [5, 6].

Frailty is a condition of high vulnerability following the deterioration of diverse organs or systems, and is often associated with poor health outcomes [7]. Although frailty is largely described in the elderly, an increased possibility of frailty is observed in chronic conditions as well as in musculoskeletal disorders [7, 8]. Frailty is associated with disease-related characteristics, comorbidities, and therapies in rheumatic diseases. In addition, frailty values may be helpful clinical- evaluation instruments for estimating unfavorable outcomes [9, 10].

The Systemic Lupus International Collaborating Clinics-Frailty Index (SLICC-FI) consist of disease activity, organ damage, comorbidities, and HRQL assessments and is generated and validated in a large population, including mestizo patients [11]. In addition, the index predicts hospitalizations, organ damage, and mortality in lupus [10, 12, 13], and more recently, the SLICC-FI was related to impaired physical activity [14]. In the multicenter LUpus in MInorities, NAture versus nurture (LUMINA) cohort, including Hispanic participants, SLICC-FI was also associated with organ damage [15]. Currently, limited studies, to our knowledge, have assessed the association between frailty and osteoporosis and VF in the general population [16, 17]. However, to our knowledge, no study analyzed this association among patients with SLE. Therefore, our aim was to investigate the relationships between SLICC-FI, BMD and VF in Mestizo women with SLE.

## Patients and Methods

This is a retrospective study that included adult women who fulfilled the 1997 Revised ACR [18] or 2012 SLICC [19] classification criteria, who were recruited consecutively between 2007 and 2012 from among patients attending the Outpatient Systemic Autoimmune Disease Research Unit, Specialities Hospital, IMSS, in Puebla City, Mexico. Participants were all females, mostly mestizo (around 85%). Pregnant patients and those with renal impairment were excluded from the study. The protocol was approved by the CIBIOR Ethics Committee (R-2022-2106-011). All the patients signed an informed consent form.

### Clinical Assessment and BMD Measurements

The participants’ clinical, laboratory, and BMD measurements were collected at baseline. Disease activity was evaluated using the SLE Disease Activity Index 2000 (SLEDAI-2K) [20] and damage was evaluated using the SLICC/ACR Damage Index [21]. BMD measurements at the lumbar spine (LS) and total hip (TH) were evaluated using dual-energy X-ray absorptiometry (DXA) with Hologic densitometry equipment (Hologic QDR Explore; Hologic, Bedford, MA, USA). The coefficient of variation for the Hologic QDR DXA system was < 1.0% for BMD.

Osteoporosis and osteopenia were categorized using the World Health Organization (W.H.O.) definitions for postmenopausal participants, utilizing the following criteria: a *T*-score ranging from − 1 to − 2.5 for osteopenia and a *T*-score of ≤ − 2.5 for osteoporosis. Finally, a *Z*-score above − 2.0 comprised a low BMD for premenopausal participants, as stated by the 2019 International Society of Clinical Densitometry (ISCD) consensus [22].

### SLICC-Frailty Index

The SLICC-FI was obtained at baseline. The SLICC-FI was generated to evaluate 48 health items, 14 domains for active inflammation, 14 for damage, six for morbidities, and 14 associated with functional deterioration, health opinion, and mental health. Table S1 (Supplementary Material) presents the SLICC-FI health deficits [10, 12], and each of which was scored from 0 (complete absence) to 1 (deficit fully present) [23]. From among the complete 48 items, we covered 45; the domains excluded were complement values and anxiety, as these data were not available for all patients at baseline. In addition, because we assessed bone loss as an outcome, we excluded the item “osteoporosis.” The SLICC-FI score was estimated for each patient as the sum of their individual health deficit scores divided by the total number of deficits. The participants were classified as robust (SLICC-FI ≤ 0.03), relatively less fit (0.03 < SLICC-FI ≤ 0.21), or frail (SLICC-FI > 0.21) [14, 24].

Previous publications have shown that the predictability of frailty scores, with the exception of a few items are eliminated when a considerable number of items are incorporated (generally more than 30) [23, 25].

### Vertebral Fractures

Lateral thoracolumbar spine X-rays were obtained from all participants and at the same time Each image was assessed and scored by a trained radiologist who was blinded to the clinical information. The semi-quantitative approach of Genant was utilized to determine VF [26]. For VF categorization, a vertebral body was regarded as fractured with a reduction in height of ≤ 20%. This approach allows for the classification of VF severity, where grade 1, is > 20–25%, grade 2 is > 25–40%, and grade 3 is > 40%. Degenerative changes were not graded as VF.

### Statistical Analysis

Categorical variables were presented as percentages. Numerical variables are shown as means and standard deviations (SD) if they were normally distributed, and as medians and interquartile ranges (IQR) otherwise. The Student *t-*test or Mann–Whitney *U* test was performed for the comparison of continuous variables, whereas the chi-square test was performed for categorical variables. Spearman rank correlation coefficients were calculated to assess the relationship between SLICC-FI score and BMD measurements. Uni- and multivariate binominal models were used to evaluate associations between frailty (categorized as SLICC-FI > 0.21) and prevalent VF, adjusted for age at diagnosis, body mass index (BMI), disease duration, cumulative glucocorticoid (GCT) dose, BMD, and bisphosphonate use. Similarly, the association between frailty and low BMD (osteopenia/osteoporosis) was performed using binary logistic regression analysis, adjusted for age at diagnosis, BMI, menopause, cumulative GCT dose, and bisphosphonate use. Variables were incorporated into the multivariable analysis if they were clinically relevant or if they had *P* values < 0.10 in the univariate models. Statistical analyses were carried out using SPSS version 26.0 statistical software for Mac (IBM, Chicago IL, USA).

## Results

### Patients’ Characteristics

We included 202 women, with a mean (SD) age of 43.3 (13.6) years. At baseline, the median (IQR) disease duration was 6 years (range 3–13 years), the mean (SD) SLEDAI-2K was 3.5 (2.1), and the median (IQR) SLICC/ACR-DI was 0 (0–1). Table 1 presents the main characteristics of the females with SLE. The mean (SD) SLICC-FI score was 0.14 (0.09), and the range was 0.01–0.49. Only 11 (5.4%) patients were categorized as robust, 62 (30.7%) as relatively less fit, 84 (41.6%) as least fit, and 45 (22.3%) as frail. In the frail group, 91.7% of the patients exhibited serious limitations in vigorous activities. In contrast, in the robust group, 9% had serious limitations in vigorous activities (*p* = 0.01). Patients categorized as frail (SLICC-FI > 021) were older (mean [SD] 50.9 [13.6] years vs. 41.2 [12.9] years; *p* < 0.001), had postmenopausal status (69.8% vs. 43.2%; *p* = 0.03), longer disease duration (mean [SD] 11.5 [9.4] years vs. 7.6 [6.7] years; *p* = 0.03), a larger cumulative GCT dose (mean [SD] 28.9 [24.7] g vs. 18.9 [27.0] g; *p* = 0.03), and a lower BMD in both TH (mean [SD] 0.909 [0.159] g/cm^2^ vs. 1.029 [0.654] g/cm^2^; *p* = 0.04) and LS (mean [SD] 0.962 [0.201] g/cm^2^ vs. 1.037 [0.171] g/cm^2^; *p* = 0.015).

**Table 1:** Baseline characteristics of the SLE patients included in this study

Baseline variables	N = 202
Age, years, mean (SD)	43.3 (13.6) years
Disease duration, years, median (IQR)	6 (3–13)
BMI, kg/m^2^, mean (SD)	28.3 (16.8)
Smokers, n (%)	24 (11.8)
Postmenopausal, n (%)	99 (49.0)
Previous fractures other than VF, n (%)	18 (8.1)
SLEDAI-2 K, score, mean (SD)	3.5 (2.1)
SDI, score, median (IQR)	0 (0–1)
Glucocorticoids, ever use, n (%)	162 (80.2)
Prednisone, mg/d, mean (SD)	11.0 (8.7)
Cumulative GC, g, mean (SD)	19.6 (21.9)
Current antimalarials use, n (%)	175 (86.6)
Current immunosuppressive drug use, n (%)	81 (40.1)
Current calcium and vitamin D supplementation, n (%)	189 (93.6)
Current bisphosphonates use, n (%)	57 (28.2)
Osteoporosis, T-score < − 2.5*, n (%)	9 (9.1)
Osteopenia, T-score < − 1.5* and > − 2.5, n (%)	25 (25.3)
Low BMD, Z-score ≤ − 1**, n (%)	5 (5.9)
At least one VF, n (%)	51 (25.2%)
Number of VF, median (IQR)	1 (0–2)
SLICC-FI score, mean (SD)	0.14 (0.09)

BMD, bone mineral density; BMI, body mass index; IQR, interquartile range; SLICC-FI, Systemic Lupus International Collaborating Clinics Frailty Index; SLEDAI-2K, Systemic Lupus Erythematosus Disease Activity Index; SDI, Systemic Damage Index

^*^Only postmenopausal patients; ** only premenopausal patients

At baseline, 51 patients (25.2%) exhibited radiographic VF. Most VF cases (76%) were grade I, and the thoracic spine was the most involved location. Only 7.7% of VF were grade III. When only premenopausal women were analyzed, 15% had at least one VF. Osteopenia and osteoporosis according to the WHO criteria were found in 25.3% and 9.1% of postmenopausal patients, respectively, while low BMD was identified in only 5.9% of premenopausal women.

### Frailty and Vertebral Fractures

In the univariate analysis, VF were associated with age (OR 1.81, 95% CI 1.02–1.97; *P* = 0.045), total hip BMD (OR 0.02, 95% CI 0.01–0.44; *P* = 00.17), and SLICC-FI > 0.21 (OR 5.76, 95% CI 2.53–13.12; *P* < 0.001). The relationship between VF and SLICC-FI > 0.21 remained significative when only women who did not have bisphosphonate exposure were analyzed (OR 5.21, 95% CI 1.98–14.2; *P* < 0.001). In the multivariable analysis, BMD at the total hip (OR 0.04, 95% CI 0.002–0.69; *P* = 0.04), and frailty (OR 5.18, 95% CI 2.29–11.73) were associated with VF after adjusting for BMI, disease duration, BMD measurements, cumulative GCT dose, and bisphosphonate use. When only premenopausal patients were analyzed, frailty was associated with VF in both the univariate and multivariate analyses. Table 2 presents the associations between frailty and VF in the entire population and premenopausal patients.

**Table 2:** Associations between SLICC-FI (> 0.21) and vertebral fractures in all cohort and in those premenopausal patients

Variable	Univariate		Multivariate^a^	
	OR (95% IC)	*P*	OR (95% IC)	*p*
*Entire population*				
SLICC-FI (> 0.21)	5.76 (2.53–13.12)	< 0.001	5.18 (2.29–11.73)	< 0.001
*Premenopausal patients*				
SLICC-FI (> 0.21)	4.49 (1.24–16.19)	0.029	4.29 (1.02–17.97)	0.047

95% CI, 95% confidence interval; BMD, bone mineral density; BMI, body mass index; GCT, glucocorticoid; OR, odds ratio; SLICC-FI, Systemic Lupus International Collaborating Clinics Frailty Index

^a^Multivariate analyses were adjusted for age, BMI, cumulative GCT dose, BMD values, bisphosphonates use and disease duration

### Frailty and Bone Mineral Density

We identified a negative relationship between SLICC-FI values and BMD at the TH (*r* = − 0.20; *P* = 0.005). Similarly, a negative correlation was identified between the SLICC-FI score and BMD at the LS (*r* = − 0.28; *P* < 0.001).

Frailty was associated with low BMD in univariate analysis (OR 3.43, 95% CI 1.7–6.89; *P* = 0.001). This association persisted when the multivariable was adjusted for confounders (OR 2.92, 95% CI 1.13–7.53; *P* = 0.02).

## Discussion

This study analyzed the associations between frailty and VF and BMD measurements in Mestizo females with SLE. Frailty was assessed using the new tool developed and validated for SLE patients [12, 23], and VF were evaluated using a standardized method as suggested by Genant [26]. We identified a strong independent association between frailty and prevalent VF. This association was also found in the group of premenopausal patients. Although we observed negative correlations between the SLICC-FI values and BMD measurements, these relationships disappeared when the multivariate analyses were performed.

Measuring frailty yields helpful information about the health condition of a patient. Frailty was determined to be a prognostic factor for poor clinical outcomes, including falls, disability, and mortality, particularly in the elderly population [27]. In our analysis, frailty was strongly associated with VF in women with SLE, even after adjusting for traditional risk determinants for VF, such as age, BMI, disease duration, cumulative GCT use, and the use of bisphosphonates. Our results, when incorporated into other studies, emphasize the relevance of frailty when assessing VF in patients with rheumatic diseases, such as rheumatoid arthritis [28, 29]. In terms of the frailty tool utilized in our study, this Index (the SLICC-FI) has been well validated and predicts several other outcomes, such as hospitalizations [13], organ damage [12], poor HRQL [14], and mortality [23].

There were some interesting differences in the risk factors for fracture between patients regarded as frail and those with less frailty or those who were not frail; the frail group was older, menopausal, had a higher cumulative GCT dose, and a lower BMD. Some of these differences have been previously identified [14]. Therefore, we consider that measuring frailty in patients with SLE may provide an essential understanding of patient’s global health condition and the risk of deficient health status, such as VF.

In our study, we found that BMD measurements correlated negatively with SLICC-FI scores, and that frailty was independently associated with low BMD (osteopenia/osteoporosis).

Our study has several limitations. This was a cross-sectional study with a small sample size, allowing us to discuss the possible relationship between frailty and low BMD and VF but not causality. Additionally, although we employed the SLICC-FI, which includes a wide spectrum of relevant items, this tool does not consider muscle strength or physical-activity levels (risk factors for low BMD and fractures). Our study included only adult female participants; therefore, studies including males are needed to explore the relationship between frailty and bone loss, because previous studies have identified sex differences in this respect [29]. Because the number of patients with robust SLICC-FI was limited (*n* = 11), comparison using this group as a reference was not possible. Our study evaluated only VF; evaluations of other fractures were not analyzed. However, since low BMD also increases the risk of non-vertebral fractures, with falls being a crucial determinant, physicians should consider frailty as a risk factor for falls and fractures, as previously described [30]. Finally, as previously mentioned, we could not assess the 48 original SLICC-FI items; however, as we could evaluate 45 items, we consider that our findings are representative of the frailty our patients underwent. This assumption is supported by previous evidence on the validity of the SLICC-FI tool utilizing fewer than the 48 items [23, 31].

With an aging population, frailty will affect an increasing number of persons, and controlling both frailty and its consequences will be a rising demand with regard to health and social systems. More insights concerning the frequency of frailty and its influence on persons with musculoskeletal diseases will reveal actions for management that bear in mind the relationships between these diseases and deficient health status. Additionally, frailty is a dynamic and potentially reversible condition, and certain deficits can be reversed [32]. Monitoring patients for frailty may increase physicians’ acknowledgment of their patients’ functionality and guide the allocation of crucial referrals for exercise strategies (based on evidence) to rehabilitate the performance of activities of daily living, grip strength, pinch strength, and dexterity [33], which may impact HRQL and prevent falls and fractures.

In conclusion, our findings indicate that frailty as defined by SLICC-FI, may be associated with low BMD and VF in women with SLE. Additional analyses evaluating the effect of frailty on the incidence of low BMD and fractures, including VF, are needed to contribute new data for potential approaches to prevent the risk of fracture and fracture-related complications and mortality in persons at higher risk.

### Supplementary Information

Below is the link to the electronic supplementary material.

Supplementary file1 (DOCX 19 KB)
