# Correction: SLICC-Frailty Index and Its Association with Low Bone Mineral Density and Vertebral Fractures in Women with Systemic Lupus Erythematosus

**DOI:** 10.1007/s00223-023-01136-6

**Published:** 2023-10-25

**Authors:** Claudia Mendoza-Pinto, Ivet Etchegaray-Morales, Pamela Munguía-Realpozo, Socorro Méndez-Martínez, Jorge Ayón-Aguilar, Francisco Arellano-Avendaño, Álvaro Joaquín Montel-Jarquín, Mario García-Carrasco

**Affiliations:** 1 Systemic Autoimmune Diseases Research Unit, Specialties Hospital UMAE- CIBIOR, Mexican Institute for Social Security, Puebla, Puebla Mexico; 2 Department of Rheumatology, Medicine School, Meritorious Autonomous University of Puebla, Puebla, Mexico; 3 Research in Health Coordination, Mexican Social Security Institute, Puebla, Mexico; 4 Health Research Medical Coordinator, Mexican Social Security Institute, Puebla Delegation, Puebla, Mexico; 5 Specialties Hospital, Centro Médico Nacional “Manuel Ávila Camacho”, IMSS, Puebla, Mexico

**Correction to: Calcified Tissue International** 10.1007/s00223-023-01117-9

The article “SLICC-Frailty Index and Its Association with Low Bone Mineral Density and Vertebral Fractures in Women with Systemic Lupus Erythematosus”, written by Claudia Mendoza-Pinto, Ivet Etchegaray-Morales, Pamela Munguía-Realpozo, Socorro Méndez-Martínez, Jorge Ayón-Aguilar, Francisco Arellano-Avendaño, Álvaro Joaquín Montel-Jarquín & Mario García-Carrasco, was originally published electronically on the publisher’s internet portal on 23 July 2023 without open access. With the author(s)’ decision to opt for Open Choice the copyright of the article changed on 23 August 2023 to © The Author(s) 2023 and the article is forthwith distributed under a Creative Commons Attribution 4.0 International License, which permits use, sharing, adaptation, distribution and reproduction in any medium or format, as long as you give appropriate credit to the original author(s) and the source, provide a link to the Creative Commons licence, and indicate if changes were made. The images or other third party material in this article are included in the article's Creative Commons licence, unless indicated otherwise in a credit line to the material. If material is not included in the article's Creative Commons licence and your intended use is not permitted by statutory regulation or exceeds the permitted use, you will need to obtain permission directly from the copyright holder. To view a copy of this licence, visit http://creativecommons.org/licenses/by/4.0/.

The original article has been corrected.

