# Supplementary material for: SLICC-Frailty Index and Its Association with Low Bone Mineral Density and Vertebral Fractures in Women with Systemic Lupus Erythematosus
Source: Calcif Tissue Int. 2023 Jul 23;113(5):475–80. doi: 10.1007/s00223-023-01117-9 (PMC10618373; doi:10.1007/s00223-023-01117-9)
Supplement: Supplementary file 1 — Supplementary file1 (DOCX 19 KB) [file 223_2023_1117_MOESM1_ESM.docx]

**Supplementary material**

**Table S1.** Systemic Lupus International Collaborating Clinics Frailty Index (SLICC-FI) health deficits.

| **Health Deficit Scoring System** | | **SCORE** |
| --- | --- | --- |
| Diabetes | No = 0; Yes = 1 | 1 |
| Malignancy | No = 0; Yes = 1 | 2 |
| Coronary artery disease | No = 0; Yes = 1 | 3 |
| Congestive heart failure | No = 0; Yes = 1 | 4 |
| Peripheral vascular disease | No = 0; Yes = 1 | 5 |
| Cerebrovascular disease | No = 0; Yes = 1 | 6 |
| Chronic kidney disease | None = 0; Stage 1 = 0.2; Stage 2 = 0.4; Stage 3 = 0.6; Stage 4 = 0.8; Stage 5 = 1 | 7 |
| Deforming or erosive arthritis | No = 0; Yes = 1 | 8 |
| Venous thromboembolism | No = 0; Yes = 1 | 9 |
| Pulmonary disease | No = 0; Yes = 1 | 10 |
| Gastrointestinal disease | No = 0; Yes = 1 | 11 |
| Ocular manifestations related to SLE | No = 0; Yes = 1 | 12 |
| SLE myocarditis/ endocarditis | No = 0; Yes = 1 | 13 |
| Cognitive impairment | No = 0; Yes = 1 | 14 |
| Seizures & seizure disorders | No = 0; Yes = 1 | 15 |
| Altered mental status | No = 0; Yes = 1 | 16 |
| Neuropathy | No = 0; Yes = 1 | 17 |
| Other neuropsychiatric manifestations | No = 0; Yes = 1 | 18 |
| Active nephritis | No = 0; Yes = 1 | 19 |
| Active nephrotic syndrome | No = 0; Yes = 1 | 20 |
| Active serositis | No = 0; Yes = 1 | 21 |
| Active inflammatory arthritis | No = 0; Yes = 1 | 22 |
| Active inflammatory rash | No = 0; Yes = 1 | 23 |
| Active mucosal ulcers | No = 0; Yes = 1 | 24 |
| Alopecia | No = 0; Yes (acute) = 0.5; Yes (chronic) = 1 | 25 |
| Active vasculitis | No = 0; Yes = 1 | 26 |
| Hematologic disorder | No = 0; Yes = 1 | 27 |
| Immunologic disorder | No = 0; Yes = 1 | 28 |
| Sjogren’s syndrome | No = 0; Yes = 1 | 29 |
| Hypothyroidism | No = 0; Yes = 1 | 30 |
| Hypertension | No = 0; Yes = 1 | 31 |
| Body mass index (BMI) | BMI 18.5 – 24.9 kg/m2 = 0; BMI 25 – 29.9 kg/m2 = 0.5; BMI ≥ 30 kg/m2 = 1 | 32 |
| Mood disorder | No = 0; Yes = 1 | 33 |
| Headache disorder | No = 0; Yes = 1 | 34 |
| Self-rated health | Excellent = 0; Very good=0.25; Good = 0.5; Fair = 0.75; Poor = 1 | 35 |
| Self-reported deterioration in health | Better or same = 0; Somewhat worse = 0.5; Much worse = 1 | 36 |
| Vigorous activities | Not limited at all = 0; Somewhat limited = 0.5; Limited a lot = 1 | 37 |
| Moderate activities | Not limited at all = 0; Somewhat limited = 0.5; Limited a lot = 1 | 38 |
| Lifting/carrying groceries | Not limited at all = 0; Somewhat limited = 0.5; Limited a lot = 1 | 39 |
| Climbing stairs | Not limited at all = 0; Somewhat limited = 0.5; Limited a lot = 1 | 40 |
| Bending, kneeling, or stooping | Not limited at all = 0; Somewhat limited = 0.5; Limited a lot = 1 | 41 |
| Walking 100 metres | Not limited at all = 0; Somewhat limited = 0.5; Limited a lot = 1 | 42 |
| Bathing or dressing | Not limited at all = 0; Somewhat limited = 0.5; Limited a lot = 1 | 43 |
| Self-rated fatigue | None = 0; A little = 0.2; Some = 0.4; Moderate = 0.6; Most = 0.8; Always = 1 | 44 |
| Self-rated pain | None = 0; Very mild = 0.2; Mild = 0.4; Moderate = 0.6; Severe = 0.8; Very severe = 1 | 45 |

Modified from Legge A, Kirkland S, Rockwood K, Andreou P, Bae SC, Gordon C, et al. Construction of a Frailty Index as a Novel Health Measure in Systemic Lupus Erythematosus. J Rheumatol. 2020;47:72-81.
